# Budget impact analysis of lens material on the posterior capsule opacification (PCO) as a complication after the cataract surgery

**DOI:** 10.1186/s12962-020-00214-y

**Published:** 2020-06-16

**Authors:** Monika Raulinajtys-Grzybek, Iwona Grabska-Liberek, Aleksandra Opala, Marta Słomka, Michał Chrobot

**Affiliations:** 1 Department of Management Accounting, Warsaw School of Economics, Warsaw, Poland; 2 Department of Ophthalmology, Centre of Postgraduate Medical Education, Warsaw, Poland; 3 Department of Neurochemistry, Mossakowski Medical Research Centre Polish Academy of Sciences, Warsaw, Poland; 4 Polish Association of Medical Coders, Kielce, Poland

**Keywords:** Posterior capsule opacification, Hydrophobic acrylic lenses, Hydrophilic acrylic lenses, Budget impact analysis

## Abstract

**Background:**

Over 300,000 cataract operations are performed in Poland every year, and the most common, late complication of cataract removal surgery is posterior capsule opacification (PCO). The risk of PCO depends on the lens material. Hydrophobic acrylic lenses cause PCO less frequently as lymphatic endothelial cells show lower affinity for the surface of the lens made of silicone. The objective of this study is to assess the economic impact of using hydrophobic acrylic lenses compared to using hydrophilic acrylic lenses for cataract treatment in the Polish inpatient and outpatient settings.

**Methods:**

A budget impact analysis (BIA) compared the economic outcomes associated with using hydrophobic acrylic lenses versus using hydrophilic lenses for patients undergoing cataract surgery. The BIA predicted annual expenses in the following scenarios: performing Nd:YAG to treat PCO within 2 and 3 years after implantation of hydrophobic or hydrophilic acrylic lenses for different lens structure. Data used to assess the frequency of PCO was determined in systematic literature review. Costs of current and predicted interventions were estimated based on average data from 19 Polish hospitals. Prices of health services were taken from official public tariff lists.

**Results:**

The use of a hydrophobic lens significantly limits the number of complications after cataract surgery relative to a hydrophilic lens. As hydrophobic lenses have a higher unit price their use increases the cost of treatment which currently is not reflected by adequate difference in price of the service. Total annual National Health Fund (NHF) expenses for 3-year follow-up model range from 139.1 million EUR to 143.1 million EUR depending on the lens structure, due to the cost of complications.

**Conclusions:**

BIA indicates the possibility of introducing surcharge for the use of hydrophobic lenses, which could increase the frequency of their use and reduce the number of complications after cataract surgery. It was estimated that total NHF expenses reach the minimum value for the surcharge at the level of 9 EUR. The surcharge of 14 EUR is the maximum value that does not increase the initial NHF expenses.

## Background

Posterior capsule opacification (PCO) is the most common late complication of cataract removal surgery with implantation of an artificial intraocular lens. PCO impairs vision in the mechanism of reduced visual acuity at far and near distances, reduced sensitivity to contrast, dazzle, perception of light scatter and double vision in one eye [1, 2]. In the 80 s and 90 s of the 20th century, the worldwide incidence of PCO in the first year after cataract surgery was in the range of 25–50% [3]. Improvement in surgery techniques, the right choice of material, size and shape of the intraocular lens has resulted in a reduced incidence of this complication [4–6]. Currently, the incidence of PCO ranges from 5 to 20% within 2–3 years of the cataract removal surgery, depending on the source [6–8].

Along with the development of microsurgery in cataract treatment, soft foldable lenses became most frequently used [9, 10]. The most frequently used soft lenses include hydrophobic acrylic lenses, hydrophilic acrylic lenses, silicone lenses and, less frequently, hydrogel lenses [11]. The type of material used affects physical properties of the lens, as well as its biocompatibility. The assessment of material biocompatibility involves determination of the severity of cellular reaction observed on the lens surface, opacity of anterior and posterior capsule, degree of capsule contraction as well as proliferation of capsular epithelium [12]. One practical aspect of biocompatibility of a given material is its potential to induce PCO.

Hydrophilic acrylic lenses are adaptable and easily implantable thanks to their slow unfolding in the capsule. The hydrophilic nature of the material facilitates proliferation and migration of lens epithelial cells (LEC), inducing PCO formation. Development of calcifications on the hydrophilic lens surface may be observed, leading to opacity, which sometimes necessitates removal of the implant [8, 10]. There are reports on low bacterial adhesion and high translucency of this material [7].

Cataract removal surgeries with implantation of a lens made of hydrophobic acryl are burdened with the lowest risk of PCO, as compared with surgeries involving lenses made of other types of material [2, 13–15]. Thanks to strong lens adhesion to the posterior capsule, LEC migration is limited, which inhibits the development of PCO [13, 16]. A sharp edge of the optical part forms a physical barrier for LEC, providing additional protection against the development of PCO. The size and shape of the implant determine the level of capsular tension and at the same time the degree of capsular adhesion to the implant [17].

A widely recognized method of PCO treatment is Nd:YAG laser capsulotomy (Neodymium: yttrium–aluminum-garnet, Nd:YAG). It involves making a small aperture in the opacified posterior capsule with the use of the neodymium YAG laser [18]. The procedure is conducted in an outpatient setting, in a specialized laboratory equipped with the Nd:YAG laser. Prior to the procedure a patient must undergo a full ophthalmological examination with special emphasis on the assessment of visual acuity and measurement of the intraocular pressure (IOP). The procedure is performed by an experienced ophthalmologist with the use of a local anesthesia preceded by pupil dilation. IOP is controlled shortly after the procedure or on the following day. The use of local anti-inflammatory drugs is recommended for 7 consecutive days.

The discussed method of PCO treatment is not free from complications. The most common complication is transient increase in IOP. According to literature, IOP increase following the procedure occurs in 15–30% cases despite taking prophylactic measures [19–21]. Literature describes single cases of lens dislocation to the vitreous chamber during the procedure of laser capsulotomy [22]. Dislocations of various degree of the artificial lens posteriorly are more common, resulting from the activity of the laser beam. Reduction in the mean value of the corneal curvature, increased depth of the anterior chamber and significant changes in the spherical equivalent of refraction (myopic shift) are typical of the early postoperative period [23, 24]. Damage to the surface area of the optical lens (pitting) resulting from the activity of the laser beam is described in literature as occurring in 9.4–19.8% cases. [25–27]. Less common complications of laser capsulotomy include chronic uveitis, occurring in 0.4–0.7% cases within 6 months of the procedure and glaucoma occurring in 1.34% cases [19]. Rare but the most serious complications of Nd:YAG capsulotomy include rhegmatogenous retinal detachment, cystoid macular edema and chronic intraocular inflammation caused by release of microorganisms from the capsule to the vitreous chamber [18, 28]. There are reports of single cases of Nd:YAG capsulotomy complications in the form of pupillary block, macular hole, hemorrhage into the vitreous chamber and secondary closure of the posterior capsular aperture [28]. Literature reports emphasize limited access to the procedure in developing countries as well as additional burden for patients and healthcare systems related to the treatment of complications [2, 19].

Available research shows that using hydrophobic lenses provides net savings to the public payer [29–31]. Budget impact analyses show lower number of post-cataract complications when using hydrophobic lenses and lower national healthcare costs. They take the payer perspective. This study includes the microeconomic perspective of a single provider who aims at maximizing its profit.

The objective of this study was to develop an economic model to estimate the budget impact for (1) a hospital performing cataract surgeries, using hydrophobic acrylic lenses vs. using hydrophilic acrylic lenses, and Nd:YAG, as well as (2) National Health Fund that reimburses all public health services. The model primarily took into account the occurrence of PCO as a result of cataract surgery and its treatment. Costs were estimated over a 2-year and 3-year horizon for Poland. Clinical outcomes were considered in this study based on a systematic literature review of the impact of two different IOL materials (hydrophobic and hydrophilic acrylate) on the development of PCO. The model compares: (1) the unit level of costs and revenues incurred by a hospital for cataract and PCO treatment, and (2) the total NHF expenses on cataract and PCO treatment in relation to different lens structure.

## Methods

An economic model was developed in Microsoft Excel to assess the potential budget impact of using hydrophobic acrylic lenses vs. using hydrophilic acrylic during a cataract surgery on the cost of both cataract treatment and Nd:YAG (as post-cataract complication). All model inputs considered a Polish hospital perspective without social or geographical variables. Budget impact was estimated at two levels: (1) a single hospital, and (2) national-level healthcare costs. In the national-level model the budget impact was estimated over a 2- and 3-year time horizon (base year 2018), assuming different lens structure. The model was developed as per the ISPOR Budget Impact Analysis Good Practice Guidelines [32].

### Model settings

The budget impact model (BIM) predicted:

- Revenues and costs of a single cataract surgery and Nd:YAG of an average Polish-based hospital,
- National-level healthcare costs of all cataract surgeries and post-cataract Nd:YAG procedures, assuming different structure of lenses used for the cataract treatment.

The BIM incorporated only direct costs, associated with the cataract surgery and Nd:YAG. The model took into account the annual number of cataract surgeries in 2018 to estimate the total costs of the cataract treatment. This was also the basis to estimate the total number of Nd:YAG as PCO treatment. Two- and three-year incidence rates of Nd:YAG capsulotomy post-cataract surgery were estimated using weighted average of PCO rates from studies retrieved through a systematic literature search for hydrophobic acrylic lenses and hydrophilic acrylic lenses. The BIM was developed to compare the total NHF expenses under different scenarios in relation to lens structure. The initial scenario was set with 0% and 100% rates of hydrophobic and hydrophilic lens, respectively. Next scenarios included the increasing proportion of hydrophobic lens.

Financial values are presented as undiscounted costs since the focus of the analysis was the expected budget at each point. The BIA includes only direct costs resulting from the treatment of cataracts and PCO. Social costs were not included, but it should be noted that both cataract and Nd:YAG procedures result in sick leave. The BIA also does not include the cost of complications occurring after PCO due to the lack of detailed data.

### Model inputs and outcomes

All model inputs (with associated sources) are presented in Table 1.

**Table 1:** Model inputs

Variable	Values	Source
Hospital-level model		
Price of cataract surgery	432.56 EUR	Average price of B19G DRG in 2018
Price of Nd:YAG	65.12 EUR	Average price of Z58 APG in 2018
Cost of hospitalization (per patient)	152.60 EUR	Average values for cataract treatment in 19 hospitals
Cost of diagnostic procedures (per patient)	9.65 EUR	Average values for cataract treatment in 19 hospitals
Cost of cataract surgical procedure (health care personnel performing surgery + operating theatre)	87.95 EUR	Average values for cataract treatment in 19 hospitals
Cost of medical materials used for cataract treatment (excluding lenses)	137.34 EUR	Average values for cataract treatment in 19 hospitals
Cost of a hydrophilic acrylic lens	45.51 EUR	Average value taken from tender data in Poland (2018)
Cost of a hydrophobic acrylic lens	64.77 EUR	Average value taken from tender data in Poland (2018)
Duration of Nd:YAG	30 min	Expert clinical knowledge
Wage of an ophthalmologist (per hour)	16.77 EUR	Average values for ophthalmology department in 19 hospitals
Wage of a nurse (per hour)	7.20 EUR	Average values for ophthalmology department in 19 hospitals
Price of Nd:YAG laser	100,000 EUR	Average value taken from tender data in Poland (2018)
Medical materials for Nd:YAG	5.97 EUR	Average values for ophthalmology department in 19 hospitals
Mark-up of indirect costs for Nd:YAG	20%	Own estimation, expert knowledge on cost structure of ophthalmology departments
Total life-cycle of Nd:YAG laser	5 years	National tax office estimates (CIT act)
Average annual number of Nd:YAG procedures (per clinic)	137	Own calculation based on number of clinics and NHF data on number of Nd:YAG procedures
National-level healthcare cost model		
Number of cataract surgeries	313 822	NHF data (2018)
Price of cataract surgery	432.56 EUR	Average price of B19G DRG in 2018
Expected rate of complications	Depending on lens structure and time horizon	Systematic literature search
Number of Nd:YAG procedures	Depending on lens structure and time horizon	Number of cataract surgeries x expected rate of complications
Price of Nd:YAG	65.12 EUR	Average price of Z58 APG in 2018
Price of medical consultation	9.30 EUR	Average price of W01 APG in 2018
Price of diclofenac (per bottle)	4.04 EUR	Official drug price lists

#### Prices of services

In the public healthcare system cataract treatment is paid case-based. Nd:YAG is paid fee-for-service (F4S) although for some hospitals the volume of outpatient services is an indicator of performance (as in payment-for-performance (P4P) model). There is a single price of Diagnosis-Related Group B19G (Cataract Treatment) as well as for Ambulatory-Patient Group Z58 (Secondary Cataract Treatment), and these prices are equal for all hospitals having a contract with NHF.

#### Resource cost data

The hospital-level cost and revenue model includes the average resource use and unit costs of resources for the cataract treatment and Nd:YAG. The following costs were included in the analysis:

- Direct-cost components: the cost of drugs, the cost of surgical materials, the cost of diagnostic procedures, and the cost of health care personnel,
- Indirect and general costs: the cost of hospitalization, the cost of operating theatre, the general and administration cost.

The costs of hydrophilic and hydrophobic lenses were obtained from tender data in Poland. Unit costs of other resources were estimated based on the data derived from 19 hospitals that reported to the National Health Services Tariff Agency in 2016 (Table 2). Calculations were based on the mean values set after cutting off outliers based on box charts analysis (boxplot) according to the formula: $\left( {Q1\, - \,1.5\left( {Q3\, - \,Q1} \right)\, \div \,Q3\, + \,\,1.5\,\left( {Q3\, - \,Q1} \right)\,} \right),$ where Q1 is the 1st quartile and Q3 means the 3rd quartile. Due to the increase in remuneration costs in the health care sector, data was updated to 2018 values assuming the annual increase by 5%.

**Table 2:** List of hospitals that provided data on costs and use of resources

Name	Location	Type of ownership	Number of hospitalizations
Wojskowy Instytut Medyczny	Warszawa	Public	7487
Wojewódzki Szpital Podkarpacki	Krosno	Public	4373
Wojewódzki Szpital Specjalistyczny nr 5 im. św. Barbary	Sosnowiec	Public	3900
Euromedic Kliniki Specjalistyczne	Katowice	Private	3720
Szpital Uniwersytecki w Krakowie	Kraków	Public	3532
4 Wojskowy Szpital Kliniczny z Polikliniką	Wrocław	Public	3376
SPZOZ w Świdnicy	Świdnica	Public	3054
Samodzielny Publiczny Szpital Wojewódzki im. Papieża Jana Pawła II	Zamość	Public	2940
Wojewódzki Szpital Specjalistyczny im. Marii Skłodowskiej–Curie	Zgierz	Public	2745
Miejskie Centrum Medyczne	Łódź	Public	2461
Mediq	Legionowo	Private	2222
Wojewódzki Szpital Zespolony	Leszno	Public	1969
Nefrolux	Siemianowice Śląskie	Private	1772
Uniwersytecki Szpital Kliniczny	Opole	Public	1706
Wojewódzki Szpital Zespolony	Płock	Public	1529
Laguna Medical Sp z.o.o	Gdynia	Private	732
OKO.M	Wrocław	Private	702
Optegra	Poznań	Private	520
Provita	Katowice	Private	116

Source: [33]

#### The total number of cataract surgeries and Nd:YAG

Data about the total number of the cataract treatment and Nd:YAG procedures has been uploaded from the NHF databases and include only those services that are paid from public funds. Out-of-pocket payments have not been included due to the lack of such data.

Data on the clinical efficacy of different types of lenses has been retrieved through a systematic literature search. The Nd:YAG capsulotomy rates for hydrophobic acrylic lenses vs. hydrophilic acrylic lenses have been compared based on research published in Embase, Medline, Medline in-process and Cochrane databases. The comparison included only research that compared a hydrophobic acrylic lens vs. a hydrophilic acrylic lens. 13 publications were found to be relevant [34–46]. All research was RCTs.

#### NHF expenses on cataract treatment

The following cost categories were included in national-level BIM:

- Expenses for the cataract surgery determined by multiplying the price of DRG procedure by the annual number of treatments in Poland,
- Expenses for the Nd:YAG determined by multiplying the price of APG procedure by the annual number of complications,
- Expenses for the ophthalmological consultation (follow-up appointment) on the day following the Nd:YAG determined by multiplying the price of consultation by the annual number of complications,
- Expenses for the nonsteroidal anti-inflammatory drug (NSAID) (Diclofenac) prescribed to patients after the Nd:YAG determined by multiplying the price of the drug by the annual number of complications. It is assumed that each patient acquires and uses one bottle.

The sensitivity analysis was performed to compare the total NHF expenses under different scenarios in relation to lens structure. The initial scenario was set with 0% and 100% rates of hydrophobic and hydrophilic lens, respectively. Next scenarios included an increasing proportion of hydrophobic lenses.

The structure of the model and its conformity with the Polish clinical practice was validated by the co-authors with clinical expertise.

## Results

### Clinical impact of lens material on the frequency of pco occurrence

The meta-analysis was performed by computing unadjusted relative risk (RR) using a fixed-effects model. RR for Nd:YAG was calculated along with the 95% confidence intervals (CIs). Between studies heterogeneity was analyzed using I2. I2 higher than 50% suggests heterogeneity. Publication bias was assessed graphically using a funnel plot. We also conducted an analysis stratified by the duration of follow-up (within 1 year, 2 years and 3 years respectively). All analyses were performed with RevMan Analyses Version 5.0.20 (© Nordic Cochrane Centre, Ringshopitalet 2008). For our analysis, we compared the individuals from hydrophobic acrylic group (experimental) vs hydrophilic group (control) who had the most comparable baseline risk factor profile as determined by each study.

The follow-up after 1 year was described in 5 studies [34–38]. In total 196 eyes were controlled in the hydrophobic experimental group. The incidence of Nd:YAG was reported in one study (Kugelberg 2006) for 4 eyes (2.0%). In the hydrophilic control group 194 eyes were controlled and 14 cases of Nd:YAG were reported (7.2%). The pooled RR was 0.35 (95% confidence interval: 0.13–0.91). The use of hydrophobic lenses reduces the risk of capsulotomy in the first year by 5.2% compared to hydrophobic lenses.

The follow-up after 2 years was described in 8 studies [37, 39–45]. In total 408 eyes were controlled in the experimental group with 20 reported events of Nd:YAG (4.9%) and 407 eyes with 90 events of Nd:YAG in the control group (22%). RR corresponding to beneficial effects of experimental group was 0.24 (95% confidence interval: 0.15–0.37). The use of hydrophobic lenses reduces the risk of capsulotomy within 2 years after the surgery by 17% compared to hydrophobic lenses.

The follow-up after 3 years was described only in 3 studies [35, 45, 46]. In total 150 eyes were controlled in the experimental group with 22 reported events of Nd:YAG (14%) and 150 eyes with 45 events of Nd:YAG in the control group (30%). RR corresponding to beneficial effects of experimental group was 0.51 (95% confidence interval: 0.34–0.76). The use of hydrophobic lenses reduces the risk of capsulotomy within 3 years after the surgery by 15% compared to hydrophobic lenses.

The use of a hydrophobic lens significantly reduces the number of complications after cataract surgery in relation to a hydrophilic lens.

### Hospital’s cost and revenue calculation

The reimbursement of ophthalmic services in Poland is a combination of case-mix with a budget cap, fee for service (F4S) and fixed budget, which includes elements of pay for performance (P4P).

#### Cost and revenue model—cataract surgery

The cataract surgery is reimbursed on the case-mix basis, which means that a hospital’s income depends on the number of services provided. At present in Poland the lens material is not a factor allowing qualification for another DRG or level of reimbursement. It is pointed out that the case-mix system allows limiting the drawbacks of other reimbursement systems, such as the lack of incentives to optimize the treatment process or the adverse selection [47–49]. It only works if it is built properly so that the groups are homogeneous both economically and medically. Otherwise cream-skimming might occur [50, 51]. The problem will intensify if the same DRG can be reported for cases which costs differ significantly.

Table 3 presents the income and costs related to the cataract surgery performed in two variants: using a hydrophilic and hydrophobic lens. The course of the service is identical and the only difference in the level of costs is related to the price of the lens.

**Table 3:** Unit costs and revenues of cataract surgery (in EUR)

	Treating cataract using hydrophilic lens	Treating cataract using hydrophobic lens
Income	432.56	432.56
Costs:	433.05	452.32
Hospitalization	152.60	152.60
Diagnostic procedures	9.65	9.65
Surgical procedure	87.95	87.95
A lens	45.51	64.77
Other medical materials	137.34	137.34
Result	−0.49	−19.76
Profitability	0%	−5%

Source: Own calculation

When calculating the cost of the cataract treatment using average costs reported by the analyzed hospitals, the cataract treatment is at the break-even point only when using a hydrophilic lens. The use of a hydrophobic lens generates a financial loss for the hospital. The difference is 19.27 EUR and results solely from the difference in the price of the lens. The use of hydrophilic lenses results in lower total cost of the service.

#### Cost and revenue—Nd:YAG

Outpatient procedures, such as Nd:YAG, can be purchased by the NHF either from hospitals that are included in the “hospital network” or other clinics. In the first case, the hospital receives a fixed budget for the whole year, and should provide all specified services, including Nd:YAG. The budget size depends, inter alia, on the quality indicators, which is part of the P4P system. Research carried out in the outpatient sector indicates that P4P can be effective in affecting health care professionals’ behavior [52, 53]. One of the performance indicators is the dynamics of the increase in the number of outpatient services. In the second case, Nd:YAG is financed on the F4S basis. Both reimbursement methods are an incentive for providers to maximize the number of services.

Nd:YAG is a profitable service (Table 4). Maximizing the number of Nd:YAGs increases the total net income of the provider.

**Table 4:** Unit costs and revenues of Nd:YAG (in EUR)

	Description	Nd:YAG
Income		65.12
Costs		62.07
Ophthalmologist	30 min. x 16.77 EUR/h	8.39
Nurse	30 min. x 7.20 EUR/h	3.60
YAG laser	100 k EUR/5 years/137 procedures	33.95
Medical materials (anesthesia)		5.97
Other costs	20% mark-up on other costs	10.16
Result		3.05
Profitability		5%

Source Own calculation

The use of hydrophilic lenses leads to a higher net financial result of a hospital than the use of hydrophobic lenses in both areas—the cataract and the PCO treatment.

### National-level health care costs

The BIA model was quantified based on the previously mentioned data sources. The analysis compared the initial scenario with 0 and 100% rates of hydrophobic and hydrophilic lens respectively with the following rates of hydrophobic to hydrophilic: 20%/80%, 40%/60%, 60%/40%, 80%/20%, 100%/0%. The calculations were performed for the capsulotomy risk rates after 2-year and 3-year period taking the expected rates of complications: 4, 9% vs. 22% for the 2-year and 14% vs. 30% for the 3-year follow-up for the hydrophobic and hydrophilic lens, respectively.

**Fig. 1:**
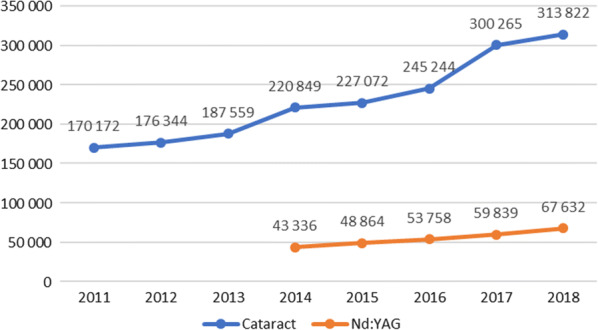
Number of cataract treatments and Nd:YAG in Poland. Source Own work based on NHF data

**Fig. 2:**
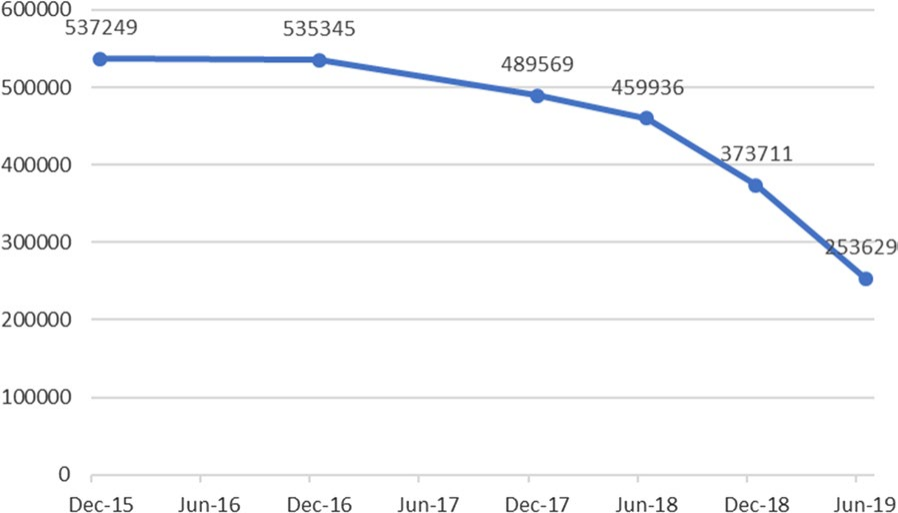
Number of patients waiting for the cataract treatment in Poland. Source Own work based on NHF data

The number of cataract treatment has been growing rapidly in Poland and in 2018 amounted to 313,822 (Fig. 1). This value is expected to stabilize as the amount of patients waiting for the treatment is declining (Fig. 2). The average price paid by NHF for the cataract treatment is 432.56 EUR and is not related to the type of lens implanted.

**Fig. 3:**
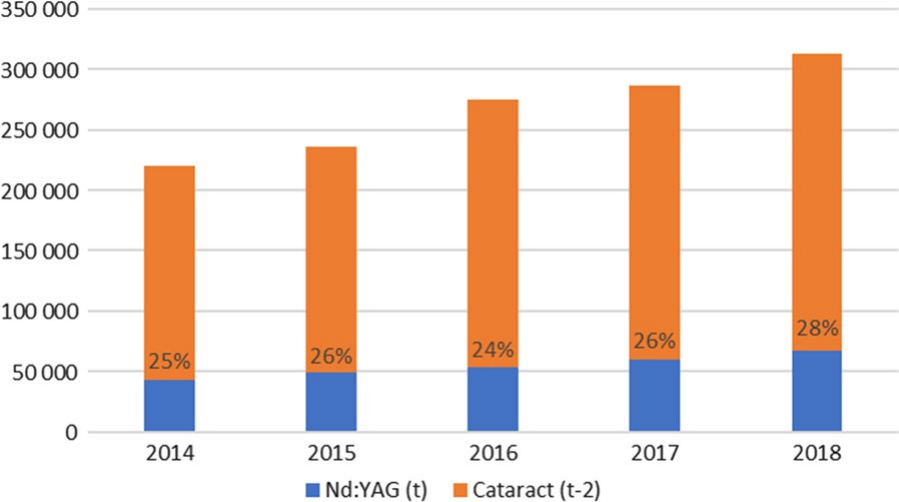
The ratio of the number of Nd:YAG procedures to the number of cataract surgeries. Source Own work based on NHF data

Currently the number of Nd:YAG is increasing proportionally to the number of cataract treatment procedures (Fig. 1). The current structure of lenses used in Poland is not available but it can be estimated that the ratio of the number of Nd:YAG procedures in year t_0_ to the number of cataract surgeries in year t_-2_ is about 25% (Fig. 3).

Considering 4, 9% and 22% of complications within 2 years after using hydrophobic and hydrophilic lens, respectively, the total annual NHF budget for treating cataract and PCO will vary from 186.3 million EUR to 190.5 million EUR depending on the lens structure (Table 5). This shows a total saving of 4.2 million EUR between a scenario of using hydrophobic acrylic lenses only and a scenario of using hydrophilic acrylic lenses only. Dividing the total savings per the total number of cataract surgeries shows a unit saving of approx. 13.42 EUR per one cataract procedure. Considering 14 and 30% of complications within 3 years after using hydrophobic and hydrophilic lens, respectively, the total annual NHF budget for treating cataract and PCO will vary from 188.6 million EUR to 192.5 million EUR depending on the lens structure (Table 5), showing a total savings of about 3.9 million EUR and a unit saving of 12.55 EUR per a cataract procedure. Greater variations in the total expenses are reported for 2-year complications due to higher reduction in capsulotomy risk observed.

**Table 5:** National-level budget impact analysis (in EUR)

	2-year follow-up					
Lens structure (hydrophobic/hydrophilic)	0%/100%	20%/80%	40%/60%	60%/40%	80%/20%	100%/0%
Expected rate of complications	22.00%	18.58%	15.16%	11.74%	8.32%	4.90%
Cataract surgery	1,35,746,844	1,35,746,844	1,35,746,844	1,35,746,844	1,35,746, 844	1,35,746,844
Nd:YAG	4,495,683	3,796,808	3,097,934	2,399,060	1,700,185	1,001,311
Consultation	6,42,240	5,42,401	4,42,562	3,42,723	2,42,884	1,43,044
Diclofenac	2,78,893	2,35,538	1,92,183	1,48,827	1,05,472	62,117
Total	1,41,163,660	1,40,321,592	1,39,479,523	1,38,637,454	1,37,795,386	1,36,953,317
	3-year follow-up					
Lens structure (hydrophobic/hydrophilic)	0%/100%	20%/80%	40%/60%	60%/40%	80%/20%	100%/0%
Expected rate of complications	30.0%	26.80%	23.60%	20.40%	17.20%	14.00%
Cataract surgery	1,35,746,844	1,35,746,844	1,35,746,844	1,35,746,844	1,35,746,844	1,35,746,844
Nd:YAG	6,130,476	5,476,559	4,822,641	4,168,724	3,514,806	2,860,889
Consultation	8,75,782	7,82,366	6,88,949	5,95,532	5,02,115	4,08,698
Diclofenac	3,80,308	3,39,742	2,99,176	2,58,610	2,18,044	1,77,477
Total	1,43, 133,411	1,42,345,511	1,41,557,610	1,40, 769,710	1,39, 981,809	1,39,193,909

Source Own calculation

Expenses are incurred mainly due to the cataract treatment. Expenses related to Nd:YAG, consultation and drug application range from 1.2 to 5.4 million EUR in a 2-year model and 3.4 to 7.4 million EUR in a 3-year model and the most important category of these expenses are the costs of Nd:YAG. The model does not include additional expenses incurred as a result of Nd:YAG complications, which would increase the actual level of expenses. The findings from these analyses suggest that variations in the lens structure in different scenarios can influence the model results.

## Discussion

Initial analysis of the BIA shows that the lens structure of 100 and 0% of hydrophobic and hydrophilic lens, respectively, minimizes total public expenses. We have further verified whether introducing a surcharge paid to hospitals when hydrophobic lens is implemented could lead to the increase in the share of hydrophobic lenses and provide lower total NHF expenses than in the initial scenario. Calculations have been done for a 2-year assumption of capsulotomy risk. 2-year values provide similar results to 3-year values, but have been set based on the bigger number of publications and therefore are treated as more reliable.

**Fig. 4:**
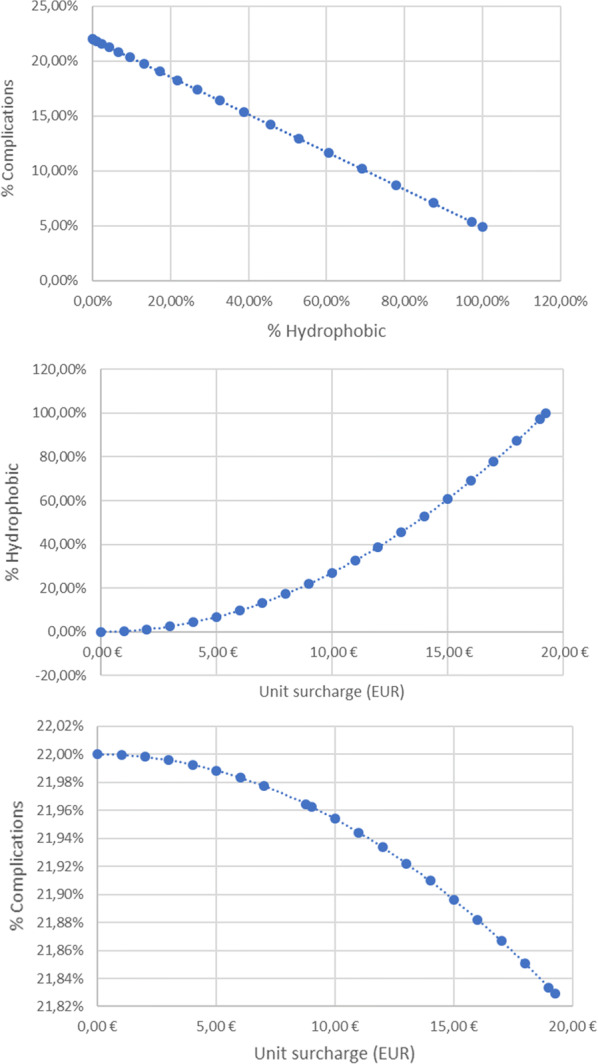
The relationship between the complications, the use of the hydrophobic lens and the level of the surcharge. Source Own work

A model was created showing the relationship between the number of complications, the share of hydrophobic lenses and the unit surcharge (Fig. 4). It was assumed that:

- There is a linear relationship between the percentage of complications and the share of hydrophobic lenses such that for 0% hydrophobic lenses complications occur after 22% phacoemulsification procedures, and for 100% hydrophobic lenses complications occur after 4.9% phacoemulsification procedures,
- Hospitals are economically rational and there is a relationship between the amount of surcharge and the share of hydrophobic lenses described by the quadratic function such that 0% of hydrophobic lenses are used for the 0 EUR surcharge, and 100% of hydrophobic lenses are used for the surcharge of 19.27 EUR. The function is:

$$\% \,Hydrophobic\, = \,0.002695\, \times \,\left( {Unit\,surch\arg e} \right)^{2} .$$

Based on the two previous assumptions, it was assumed that there is a relationship between the complication rate and the amount of the surcharge. All functions are presented below.

$$\% \,Complications\, = \, - 0.171\,\, \times \,\% Hydrophobic\, + \,0.22.$$

$$\% \,Hydrophobic\, = \,0.002695\,\, \times \,\left( {Unit\,surch\arg e} \right)^{2} .$$

$$\% \,Complications\, = \, - 0.000461\, \times \,\left( {Unit\,surch\arg e} \right)^{2} \, + \,0.22.$$

A model of the total NHF expenses, assuming that the payment is paid when using a hydrophobic lens (see Table 6). The total number of cataract procedures have been set as 313,822 (2018 value for Poland). Total surcharge expenses as well as the total number of complications depend on the share of hydrophobic lenses, which in turn depends on the level of the unit surcharge. For example, for a unit surcharge of 1 EUR:

**Table 6:** The impact of surcharge on total NHF costs of cataract and PCO treatment

Unit surcharge	Rate of complications	Cataract	Total surcharge	Nd:YAG	Consultation	Diclofenac	Total cost
0,00 €	22.00%	1,35,746,844	–	4 ,495,940	6,42 ,080	1,71 ,912	1,41,056,775
1,00 €	21.95%	1,35,746,844	846	4,48,522	6,40,735	1,71,552	1,41,04,498
2,00 €	21.82%	1,35,746,844	6766	4,45,268	6,36,700	1,70 471	1,41,019,049
3,00 €	21.59%	1,35,746,844	22,835	4,411,178	6,29,975	1,68,671	1,40,979,503
4,00 €	21.26%	1,35,746,844	54,128	4,345,253	6,20,560	1,66,150	1,40,932,935
5,00 €	20.85%	1,35,746,844	1,05,719	4,260,491	6,08,455	1,62,909	1,40,884,419
6,00 €	20.34%	1,35,746,844	1,82,683	4,156,894	5,93,660	1,58,948	140,839,029
7,00 €	19.74%	1,35,746,844	2,90,094	4,034,461	5,76,175	1,54,266	1,40,801,840
8,00 €	19.05%	1,35,746,844	4,33,027	3,893,192	5,55,999	1,48,864	1,40,77,927
*9,00 €*	*18.27%*	*1,35,746,844*	*6,16,556*	*3,733, 087*	*5,33,134*	*1,42,742*	*1,40,772,364*
10,00 €	17.39%	135,746,844	8,45,756	3,554,146	5,07,579	1,35,900	1,40,790,226
11,00 €	16.42%	1,35,746,844	1,125,701	3,356,370	4,79,334	1,28,338	1,40,836,587
12,00 €	15.36%	1,35,746 ,844	1,461,466	3,139,758	4,48,399	1,20,055	1,40,916,522
13,00 €	14.21%	1,35,746,844	1,858,125	2,904,309	4,14,774	1,11,052	1,41,035,105
14,00 €	12.97%	1,35,746,844	2,320,754	2,650,025	3,78,459	1,01,329	1,41,197,411
15,00 €	11.63%	1,35,746,844	2,854,426	2,376,905	3,39,454	90,886	1,41,408,515
16,00 €	10.20%	1,35,746,844	3,464,216	2,084,949	2,97,758	7,9,722	1,41,673,490
17,00 €	8.68%	1,35,746,844	4,155,198	1,774,158	2,53,373	6,7,839	1,41,997,412
18,00 €	7.07%	1,35,746,844	4,932,448	1,444,530	2,06,298	55,235	1,42,385,355
19,00 €	5.36%	1,35,746,844	5,801,039	1,096,067	1,56,533	41,910	1,42,84,393
19,26 €	4.90%	1,35,746,844	6,045,088	1,001,368	1,43,009	38,289	1,42,974,598

Source: Own calculation

Italic values indicate the minimum of the function of total cost

- 0.27% share of hydrophobic lenses and 21.95% PCO complications,
- Total surcharge at the level of 0.21% out of 313,822 procedures × 1 EUR = 846 EUR,
- Nd:YAG expenditure, control visits and Diclofenac on the level of 5298 808 EUR: 0.21% out of 313,822 procedures x (65.12 EUR + 9.30 EUR + 2.49 EUR).

**Fig. 5:**
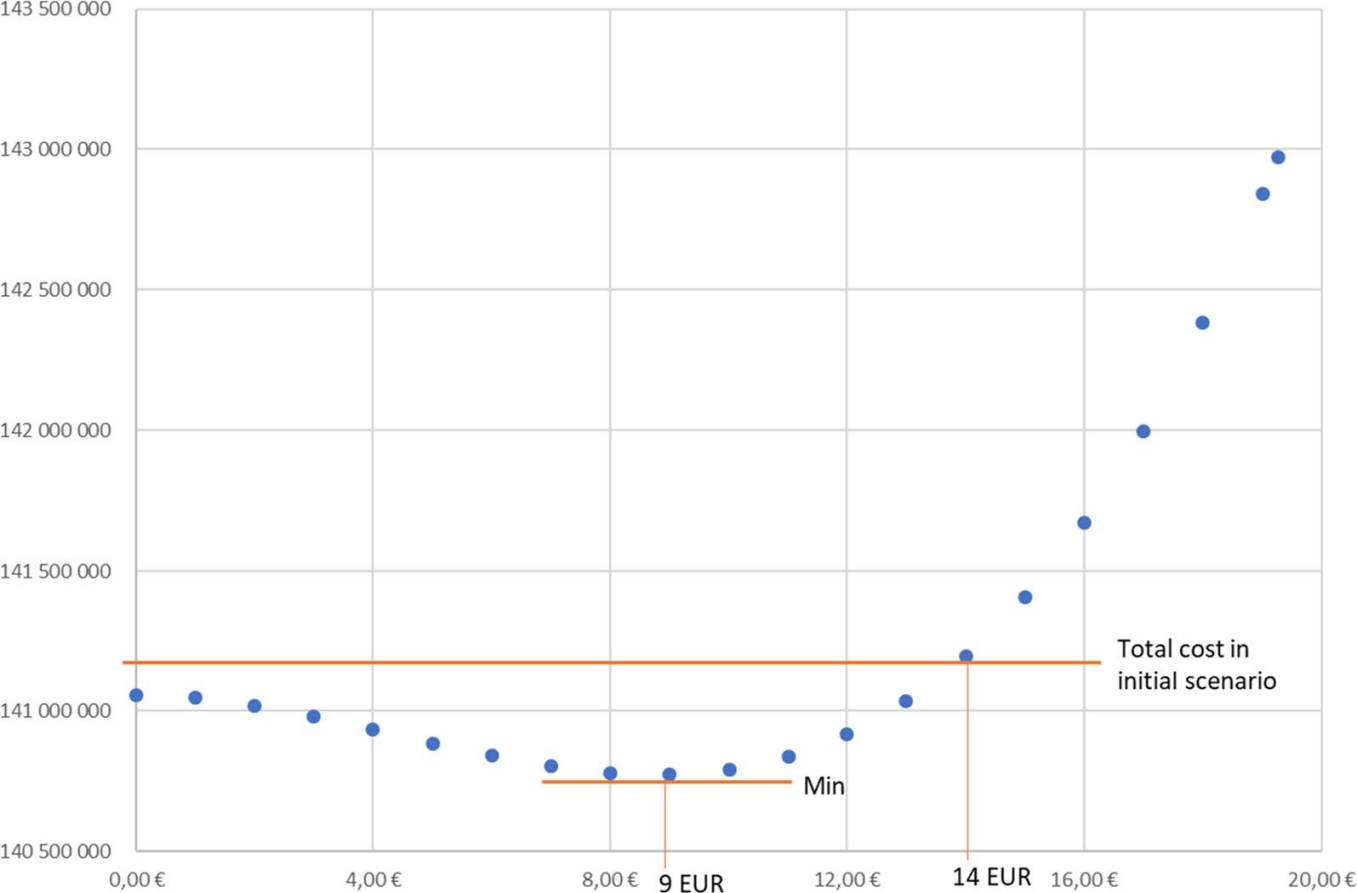
Total social costs depending on the amount of the surcharge. Source Own work

On the basis of empirical data a graph was constructed showing the relation of the total social costs from the unit surcharge paid for the implantation of the hydrophobic lens (Fig. 5).

This function achieves a minimum for a unit surcharge of approx. 9 EUR/procedure. For such a unit surcharge the total NHF costs will amount to 140.8 million EUR. The total surcharge will amount to 0.6 million EUR while costs related to PCO should decrease from the initial level of 5.3 million EUR to 4.4 million EUR. For this level of payment 22% of the implanted lenses will be hydrophobic.

The maximum level of the surcharge, which does not increase the initial total NHF expenses is approx. 14 EUR/procedure. For such a unit surcharge the total costs will amount to 141.2 million EUR and remain almost unchanged. The total subsidy will amount to 2.3 million EUR while costs related to PCO should decrease to 3.1 million EUR. For this level of payment 53% of the implanted lenses will be hydrophobic.

## Conclusion

The objective of this study was to assess the economic impact of using hydrophobic acrylic lenses compared to using hydrophilic acrylic lenses for cataract treatment in the Polish inpatient and outpatient settings. We took two perspectives—of a single hospital as well as the National Health Fund that reimburses all public health services.

Hydrophobic lenses have a higher unit price their use increases the cost of treatment which currently is not reflected by adequate difference in price of the service. Therefore the hospital’s income is higher when using the hydrophilic lenses. There are also financial incentives to maximize the number of Nd:YAG procedures.

At the same time the use of a hydrophobic lens significantly limits the number of complications after cataract surgery relative to a hydrophilic lens. Total annual National Health Fund (NHF) expenses for 3-year follow-up model range from 139.1 million EUR to 143.1 million EUR depending on the lens structure, due to the cost of complications. The lowest value is achieved by maximizing the use of hydrophobic lenses.

In the discussion we tried to analyze if the surcharge for the use of hydrophobic lenses can be included in the model. Such a surcharge could increase the frequency of the use of hydrophobic lenses and reduce the number of complications after cataract surgery. It was estimated that total NHF expenses reach the minimum value for the surcharge at the level of 9 EUR. The surcharge of 14 EUR is the maximum value that does not increase the initial NHF expenses.

## Limitations of the study

The study assumes that all hospitals will strive to achieve the economic result and that their costs are equal to model values. Underestimation of hospital costs and an additional motivating factor for the implementation of Nd:YAG resulting from the P4P or F4S system may consequently cause different behavior of service providers.

The authors assumed that the relationship between the amount of surcharge and the share of hydrophobic lenses is described by the quadratic function. If the behavior of provided is to be described by other function, different results are to be expected.

The topic of indirect costs has been omitted. Patients undergoing cataract as well as PCO tend to be on sick leave, and there is a share of patients who are not retired, which has a consequence for the GDP. Additionally, patients waiting for Nd:YAG might also be less effective at work. The expenditure for the treatment of complications after laser capsulotomy was also omitted although the presented studies indicate that these expenses concern a significant percentage of treatments.

## Data Availability

The datasets during and/or analysed during the current study available from the corresponding author on reasonable request.
